# Mitochondrial membrane remodeling in stress adaptation: Lipid control of organelle quality

**DOI:** 10.1002/pro.70703

**Published:** 2026-07-09

**Authors:** Lena J. Reichert, F.‐Nora Vögtle, Carlotta Peselj

**Affiliations:** ^1^ Center for Molecular Biology of Heidelberg University (ZMBH), DKFZ‐ZMBH Alliance Heidelberg Germany; ^2^ Network Aging Research Heidelberg University Heidelberg Germany; ^3^ CIBSS—Centre for Integrative Biological Signalling Studies University of Freiburg Freiburg Germany

**Keywords:** cardiolipin, ER–mitochondria crosstalk, mitochondrial membrane remodeling, mitochondrial protein biogenesis, mitochondrial unfolded protein response (UPR^mt^), organelle stress signaling

## Abstract

Mitochondria respond to proteotoxic stress through the mitochondrial unfolded protein response, traditionally viewed as a transcriptional program that restores proteostasis by inducing chaperones and proteases. Emerging evidence indicates that mitochondrial membrane remodeling constitutes an additional adaptive component of this response. Regulated changes in mitochondrial lipid composition, particularly involving the signature phospholipid cardiolipin, support mitochondrial function during stress by stabilizing protein import machineries, promoting mitochondrial protein biogenesis, and facilitating recovery from dysfunction. In addition, stress originating in other organelles, especially the endoplasmic reticulum, reshapes mitochondrial membranes through altered lipid biosynthesis, inter‐organelle lipid trafficking, and stress signaling pathways. These findings suggest that mitochondrial membrane remodeling represents a regulatory layer of organelle quality control integrated within interconnected stress response networks and may provide new opportunities to enhance mitochondrial resilience in disease.

## 1 INTRODUCTION

Eukaryotic cells are highly compartmentalized systems in which specialized organelles coordinate distinct processes. This spatial organization enhances reaction efficiency, enables regulatory precision, and protects the cell from potentially harmful biochemical reactions. To preserve organelle integrity, cells have evolved dedicated quality control pathways that detect dysfunction and activate adaptive responses to restore homeostasis. A central principle of organelle quality control is the unfolded protein response (UPR), which is triggered by proteotoxic stress and induces protective transcriptional programs. The best‐characterized examples are the UPR of the endoplasmic reticulum (UPR^ER^) and the mitochondrial unfolded protein response (UPR^mt^). Both pathways transmit signals from the stressed organelle to the nucleus, resulting in changes in nuclear gene expression that enhance protein folding and degradation capacity through the upregulation of molecular chaperones and proteases (Cox et al., 1993; Kozutsumi et al., 1988; Mori et al., 1992; Nargund et al., 2012; Vögtle, 2021; Zhao et al., 2002).

Importantly, studies of the ER have established that adaptive stress responses extend beyond proteostasis. During UPR^ER^ activation in yeast, the ER undergoes extensive membrane remodeling, including organelle expansion that increases its folding capacity and promotes recovery from stress (Bernales et al., 2006; Schuck et al., 2009). This adaptation is driven by transcriptional changes that upregulate lipid biosynthesis pathways, establishing a direct mechanistic link between stress signaling and membrane biogenesis. Over the past decades, a strong connection between ER stress signaling and lipid metabolism has been demonstrated (Jonikas et al., 2009; Pineau et al., 2009; Promlek et al., 2011; Schuck et al., 2009; Surma et al., 2013; Thibault et al., 2012; Travers et al., 2000). Thus, membrane remodeling is now recognized as an integral and protective component of the ER stress response.

In contrast, the UPR^mt^ has long been viewed primarily as a transcriptional pathway that enhances mitochondrial proteostasis through increased production of chaperones and proteases. Severe mitochondrial dysfunction ultimately results in large‐scale membrane rearrangements associated with mitophagy. However, whether earlier adaptive changes in mitochondrial membrane composition occur and contribute to stress adaptation remains largely unknown.

Membrane remodeling provides a powerful mechanism to modulate organelle function. Changes in lipid composition alter physicochemical membrane properties—including fluidity, permeability, phase behavior, curvature, and surface charge—which in turn influence the activity, stability, and organization of membrane‐associated protein complexes (Bigay & Antonny, 2012; Ernst et al., 2018; Harayama & Riezman, 2018; Sezgin et al., 2017). Even subtle changes in lipid species can therefore have profound functional consequences, suggesting that regulated lipid remodeling may contribute to cellular protection during mitochondrial stress prior to the onset of irreversible damage.

A central player in mitochondrial membrane organization is the signature phospholipid cardiolipin (CL). CL consists of four acyl chains and two phosphate headgroups linked by a glycerol moiety and is predominantly localized to the inner membrane (IM), where it represents a major fraction of mitochondrial phospholipids, and to a minor extent to the outer membrane (OM) (Vögtle et al., 2015; Zinser et al., 1991). Due to its unique biophysical properties, CL forms strong non‐covalent interactions with mitochondrial membrane proteins and plays essential roles in maintaining mitochondrial structure and function. In particular, CL stabilizes respiratory chain complexes and supports their organization into higher‐order assemblies, thereby contributing to efficient oxidative phosphorylation and IM architecture (Calzada et al., 2019; Claypool et al., 2008; Fry & Green, 1981; Mileykovskaya & Dowhan, 2009; Pfeiffer et al., 2003; Prola et al., 2021; Sharpley et al., 2006; Shinzawa‐Itoh et al., 2007; Zhang et al., 2002, 2005). Through lateral segregation of large protein complexes, CL also helps shape IM organization. In addition to its role in oxidative phosphorylation, loss of CL also disrupts mitochondrial protein translocases and results in defects in mitochondrial protein import, including impaired biogenesis of OM proteins (Gebert et al., 2009). Given these central roles, regulated changes in CL abundance or composition are ideally suited to serve as an adaptive mechanism for rapidly modulating mitochondrial function during stress.

**FIGURE 1:**
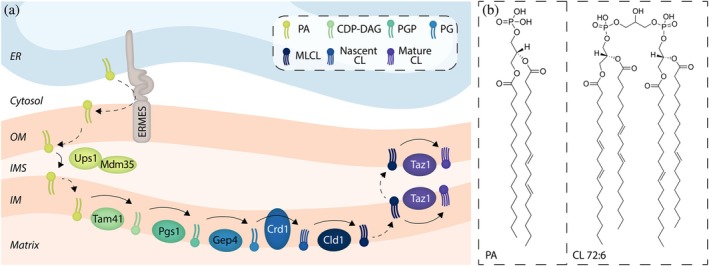
(a) Schematic overview of cardiolipin (CL) biosynthesis in mitochondria. Phosphatidic acid (PA) is transported from the endoplasmic reticulum (ER) to the outer membrane (OM), potentially via ER–mitochondria encounter structure (ERMES). PA is transferred across the intermembrane space (IMS) to the inner membrane (IM) with the help of Ups1 and Mdm35 (PRELID‐TRIAP1 in humans). In the IM, PA is converted to cytidine diphosphate‐diacylglycerol (CDP‐DAG), phosphatidylglycerolphosphate (PGP), phosphatidylglycerol (PG), and nascent CL by Tam41, Pgs1, Gep4, and Crd1, respectively (TAMM41, PGS1, PTPMT1, and CLS1/CRLS1 in humans). Nascent CL is remodeled by Cld1 (in humans, enzymes of the iPLA2 family have been implicated in this process) into monolysocardiolipin (MLCL), which can be shuttled from the IM to the OM. Taz1 converts MLCL to mature CL in the IM and OM. In humans, three enzymes have been implicated in CL remodeling: MLCLAT1, associated with the matrix‐facing side of the IM; Tafazzin, localized to the IMS‐facing side of the IM; and ALCAT1, an ER protein localized at mitochondria‐associated membranes. Dashed arrows indicate that the corresponding proteins involved in the transport process are not yet known or validated. (b) Chemical structures of PA and CL (72:6).

Understanding how such regulation could be achieved requires insight into the pathways that govern CL biogenesis and remodeling. CL is synthesized and remodeled through a multi‐step pathway that links lipid metabolism between the ER and mitochondria (Figure 1; Kagan et al., 2014; Schlame & Ren, 2009). CL biosynthesis begins with phosphatidic acid (PA), which is generated in the ER and transported to mitochondria. The mechanisms by which PA is transferred from the ER to the OM remain unresolved, although the ER–mitochondria encounter structure (ERMES) has been proposed to function either as an active lipid transporter or as a membrane contact site facilitating lipid exchange (Gallas et al., 2006; Kornmann et al., 2009; Nguyen et al., 2012; Tamura et al., 2013; Voss et al., 2012). Transport of PA from the OM to the IM is better characterized and involves the intermembrane space (IMS) protein Ups1, assisted by Mdm35, which binds and transfers PA across the IMS (Connerth et al., 2012; Potting et al., 2010; Sesaki et al., 2006; Tamura et al., 2010). Within the IM, PA is converted into cytidine diphosphate‐diacylglycerol (CDP‐DAG) by the peripheral IM protein Tam41 (Tamura et al., 2013). CDP‐DAG is subsequently used by the enzyme Pgs1 to generate phosphatidylglycerophosphate (PGP) (Chang et al., 1998). The peripherally IM‐associated protein Gep4 then dephosphorylates PGP to produce phosphatidylglycerol (PG), which serves as a key precursor for CL synthesis (Osman et al., 2010). Finally, PG and CDP‐DAG are condensed by the CL synthase Crd1 to generate nascent CL (Jiang et al., 1997). Newly synthesized CL undergoes extensive acyl chain remodeling to produce mature CL species with defined acyl chain composition. The phospholipase Cld1 removes an acyl chain from CL to generate monolysocardiolipin (MLCL) (Beranek et al., 2009), and the transacylase Taz1, localized to both the IM and OM and facing the IMS, reacylates MLCL by transferring an acyl chain from donor phospholipids. This remodeling process generates CL species with varying degrees of unsaturation that are essential for mitochondrial membrane function (Claypool et al., 2006; Gebert et al., 2009; Gu et al., 2004; Xu et al., 2003, 2006). Defects in CL synthesis and remodeling have been implicated in a plethora of severe pathologies in humans. The best‐characterized pathology is Barth syndrome, which is caused by mutations in the *TAZ* gene encoding the phospholipid transacylase required for CL remodeling (Chin & Conway, 2020; Raja et al., 2017; Schlame & Ren, 2006). In addition, impaired CL synthesis and remodeling have been linked to dilated cardiomyopathy, heart failure, and ischemia–reperfusion injury, and implicated in neurodegenerative diseases such as Parkinson's and Alzheimer's diseases.

Regulation of mitochondrial membrane composition does not occur in isolation but depends on lipid exchange with other cellular compartments. In recent years, the discovery of extensive membrane contact sites between organelles has revealed a structural basis for such inter‐organelle coordination (Scorrano et al., 2019; Voeltz et al., 2024). These contact sites enable non‐vesicular transfer of lipids and metabolites and allow rapid communication between organelles. In particular, close contacts between the ER—the major site of cellular lipid biosynthesis—and mitochondria support the exchange of lipid precursors required for mitochondrial membrane biogenesis (Kawano et al., 2018; Lahiri et al., 2014). Beyond their role in maintaining membrane homeostasis under physiological conditions, these connections raise the possibility that stress signals originating in one organelle can influence the function and membrane composition of another. Understanding how mitochondrial membranes respond to other organelle stress, and whether such changes represent protective adaptations, has therefore emerged as an important open research question.

In this review, we discuss emerging evidence that mitochondrial membrane remodeling—particularly changes in CL abundance and composition—constitutes an underappreciated but essential protective branch of the UPR^mt^. We highlight how stress‐induced lipid remodeling stabilizes mitochondrial protein import machineries, supports bioenergetic function, and facilitates recovery from proteotoxic stress. Furthermore, we examine how stress signaling from other organelles, especially the ER, reshapes mitochondrial membrane composition, emphasizing that organelle stress responses operate within interconnected adaptive organelle networks.

## 2 MITOCHONDRIAL LIPID REMODELING AS A PROTECTIVE BRANCH OF THE UPR ^mt^

Protective programs that monitor mitochondrial function and respond to mitochondrial defects have been demonstrated across multiple species, including yeast, nematodes, mice, and human cells (Andreasson et al., 2019; Charmpilas et al., 2025; Eisenberg‐Bord & Schuldiner, 2017; Melber & Haynes, 2018; Münch, 2018; Vögtle, 2021). The UPR^mt^ can be induced by diverse forms of mitochondrial dysfunction, including defects in oxidative phosphorylation, dissipation of the mitochondrial membrane potential, impaired protein import, depletion of mitochondrial DNA, inhibition of mitochondrial translation, or reduced protein folding capacity within the matrix. These perturbations are communicated to the nucleus, where they trigger a conserved transcriptional program characterized by the induction of mitochondrial chaperones and proteases, which are subsequently imported into mitochondria to restore mitochondrial proteostasis (Charmpilas et al., 2025; Münch, 2018; Vögtle, 2021). In parallel, mitochondrial stress induces a global attenuation of cytosolic translation, reducing the burden of newly synthesized proteins on the organelle (Charmpilas et al., 2025; Münch, 2018).

Initial indications that lipid biosynthesis may contribute to cellular communication during mitochondrial stress emerged approximately a decade ago with the description of a mitochondria‐to‐cytosolic stress response (MCSR) in *Caenorhabditis elegans* (Kim et al., 2016). In this study, depletion of the mitochondrial Hsp70 homolog *hsp‐6* resulted not only in the induction of canonical UPR^mt^ markers but also in the activation of components of the cytosolic heat shock response (HSR). Notably, RNAi‐mediated depletion of the other 11 members of the Hsp70 family did not elicit a comparable cross‐compartmental stress response. The MCSR required established components of the UPR^mt^ pathway, including the transcription factors DVE‐1 and ATFS‐1, as well as HSF‐1, a central regulator of the cytosolic HSR, and was reported not to arise from increased cytosolic protein aggregation.

Transcriptomic analysis revealed enrichment of genes involved in lipid biosynthetic pathways, a signature that was distinct from that observed upon isolated activation of either the UPR^mt^ or the HSR. Consistent with altered lipid metabolism, lipid droplet formation was increased. Genetic depletion of enzymes required for fatty acid biosynthesis prevented induction of the MCSR, whereas inhibition of fatty acid oxidation—which promotes fat accumulation—was sufficient to trigger the response. These observations prompted lipidomic analysis of *hsp‐6* depleted worms, which revealed increases in ether lipids, phospholipids, and precursors of phosphatidylglycerol, accompanied by reduced levels of ceramide, a class of sphingolipids consisting of a sphingoid base linked to a fatty acid. Nonyl acridine orange staining further indicated elevated levels of CL, and inhibition of CL synthesis prevented induction of the MCSR. Intriguingly, CL is an inhibitor of ceramide synthesis, and treatment with ceramides was sufficient to suppress the response.

Functionally, activation of the MCSR was associated with improved cytosolic proteostasis, reflected by reduced accumulation of proteotoxic polyglutamine (polyQ) aggregates and delayed progression of motility defects in polyQ‐expressing animals. Based on these observations, the authors proposed a model in which mitochondrial stress leads to increased CL levels, which in turn act as an initiating signal for the MCSR through inhibition of ceramide synthesis. In this model, altered lipid metabolism mediates communication between mitochondrial and cytosolic stress pathways.

While this study provides an interesting first description linking lipid biosynthesis to stress signaling during putative mitochondrial dysfunction, several aspects remain to be clarified. The mechanistic connection between hsp‐6 depletion and changes in CL levels has not yet been resolved. Moreover, because hsp‐6 has been reported to be present in small amounts in the cytosol, it remains to be fully established whether the observed cross‐compartmental effects arise exclusively from mitochondrial dysfunction or may also reflect contributions from cytosolic Hsp70 depletion. Further experimental validation will therefore be required to determine the precise causal relationships and to substantiate the proposed signaling model. Nevertheless, this work represents an early step in implicating lipid metabolism, and in particular CL, in mitochondrial stress communication.

The concept that lipid molecules may function as signaling determinants during mitochondrial stress was supported by an elegant study investigating how the protein Vms1 is selectively recruited to dysfunctional mitochondria in yeast (Nielson et al., 2017). Cytosolic Vms1 is targeted to damaged mitochondria, where it acts as an adaptor for the AAA‐ATPase Cdc48 (p97 in human cells), which extracts ubiquitylated OM proteins for degradation by the 26S proteasome (Heo & Rutter, 2011). Because Vms1 localizes specifically to damaged but not healthy mitochondria, a stress‐induced mitochondrial marker must facilitate its recruitment. To identify this signal, approximately 500 yeast mutants lacking individual non‐essential mitochondrial proteins were screened. However, none showed a substantial reduction in Vms1 localization to mitochondria. Furthermore, treatment of isolated mitochondria with proteinase K to remove surface‐exposed proteins did not impair Vms1 binding. These findings suggested that proteins were not required for recruitment, shifting attention toward mitochondrial lipids, which consist predominantly of phospholipids but also include sphingolipids and sterols.

Systematic testing of liposomes with defined lipid compositions, combined with lipid‐binding analyses and fractionation approaches, identified a specific candidate: ergosterol peroxide (EP), which was confirmed by nuclear magnetic resonance (Nielson et al., 2017). EP is an oxidized derivative of ergosterol generated non‐enzymatically in the presence of reactive oxygen species (ROS) and differs from ergosterol by the presence of an endoperoxide. Notably, EP was found to be enriched in mitochondrial membranes compared to other cellular membranes, consistent with a role in selectively recruiting Vms1 to damaged mitochondria. Liposomes derived from ergosterol‐deficient cells failed to bind Vms1, whereas the addition of EP, but not ergosterol, promoted Vms1 recruitment.

To substantiate these findings in vivo, yeast cells were exposed to ROS‐inducing conditions, including rapamycin treatment or exogenous H_2_O_2_. Both treatments increased mitochondrial EP levels and enhanced Vms1 recruitment (Nielson et al., 2017). It is noteworthy that the mechanism relies on a sterol derivative, given that ergosterol itself is more abundant in other cellular membranes. The enrichment of EP specifically in mitochondria may reflect the fact that mitochondria are a major source of ROS from the respiratory chain. In addition, higher turnover of EP in non‐mitochondrial membranes may contribute to its mitochondrial accumulation. Because the study primarily employed externally induced ROS stress, it will be important to determine whether endogenous ROS generated within the matrix or IMS by the electron transport chain similarly promote EP formation and Vms1 recruitment.

In addition to ROS, the accumulation of proteotoxic protein aggregates represents a classical trigger of the UPR^mt^, as demonstrated across multiple model systems. In yeast, a temperature‐sensitive mutation in the mitochondrial presequence protease (MPP), an essential enzyme that removes targeting sequences from precursor proteins upon their import into the matrix, leads to the rapid accumulation of unprocessed precursor proteins and the formation of proteotoxic aggregates (Poveda‐Huertes et al., 2021). This perturbation robustly induces the UPR^mt^. Because the mutant phenotype can be precisely controlled by temperature shifts, this system provides a well‐defined experimental model to monitor mitochondrial dysfunction over time and to analyze different stages of adaptive responses (Poveda‐Huertes et al., 2020, 2021).

**FIGURE 2:**
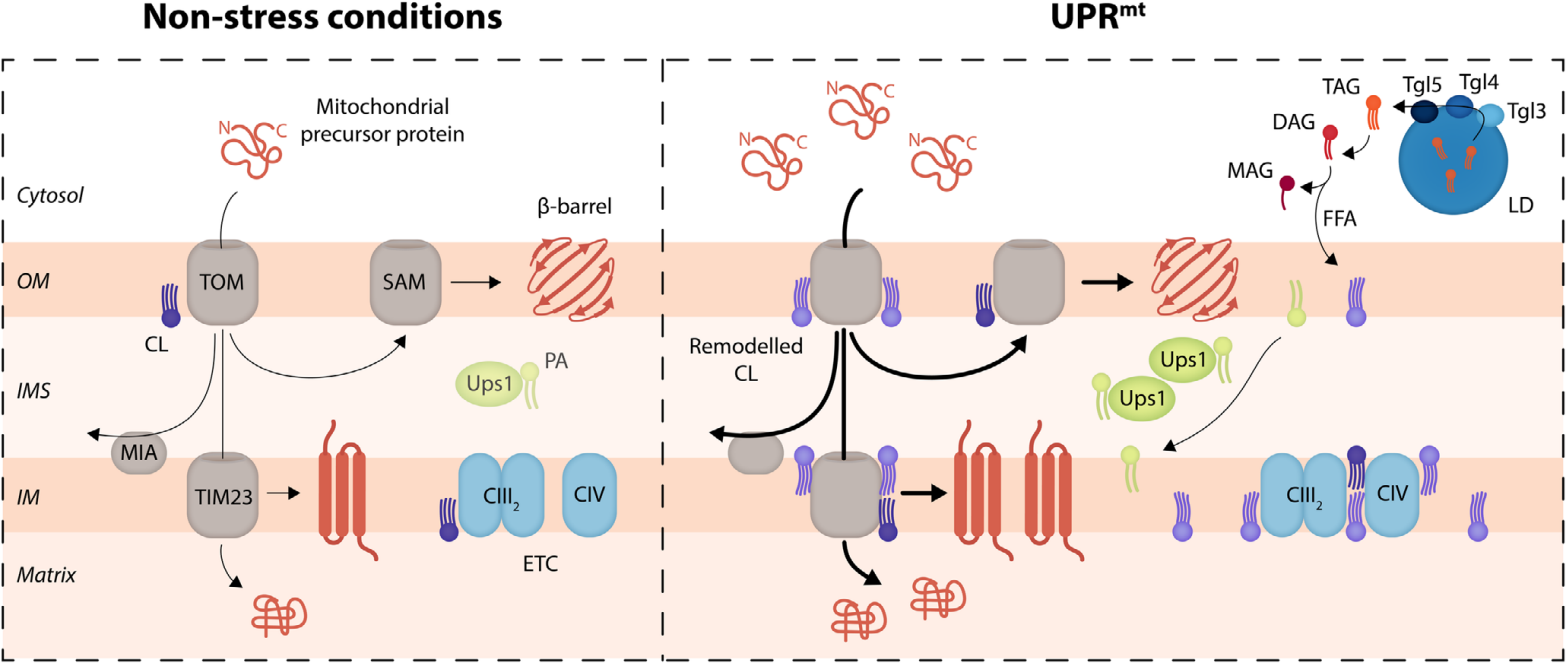
Depiction of membrane remodeling during mitochondrial unfolded protein response (UPR^mt^). Left panel: Mitochondria under non‐stress conditions. Right panel: Mitochondria following induction of UPR^mt^. Elevated Ups1 levels and the resulting increase in phosphatidic acid (PA) in the inner membrane (IM) lead to a boost in cardiolipin (CL) synthesis. Furthermore, CL undergoes remodeling, favoring longer acyl chains and a higher degree of unsaturation. CL stabilizes the mitochondrial import machineries in the inner and outer membrane and the respiratory chain complexes (electron transport chain [ETC]), leading to an increase in protein import into all mitochondrial compartments and maintenance of oxidative phosphorylation. In addition, free fatty acids (FFA) derived from lipase‐mediated triacylglycerol (TAG) hydrolysis in lipid droplets (LD) are channeled into mitochondria, where they further stimulate CL synthesis and remodeling. DAG, diacylglycerol; IMS, intermembrane space; MAG, monoacylglycerol; OM, outer membrane.

Using this approach, induction of the transcriptional nuclear response was found to be accompanied by rapid and pronounced changes in mitochondrial membrane lipid composition (Figure 2). In particular, CL levels increased, and CL underwent extensive remodeling, ultimately resulting in longer acyl chains and a higher degree of unsaturation compared with control conditions. These changes were paralleled by transient accumulation of the CL precursor CDP‐DAG, which peaked shortly after induction and was subsequently depleted, consistent with enhanced CL synthesis. In addition, levels of the CL biogenesis factors Ups1 and Pgs1 were increased, further supporting activation of the CL biosynthetic pathway (Poveda‐Huertes et al., 2021).

Strikingly, the changes in CL abundance and composition coincided with alterations in mitochondrial protein import. Increased biogenesis of precursor proteins into all mitochondrial compartments was observed using authentic substrates (Poveda‐Huertes et al., 2021). Mitochondrial protein import is mediated by translocases in the OM and IM, and previous work has demonstrated that loss of CL results in defects in protein import (Gebert et al., 2009). Consistent with a direct effect of lipid remodeling on the import machinery, analysis of the extractability of translocase complexes from the lipid bilayer revealed altered membrane association upon UPR^mt^ induction, indicating changes in the surrounding lipid environment. These effects were dependent on CL, as deletion of Crd1 restored translocase extractability to control levels. Importantly, the functional relevance of CL remodeling was supported by growth analyses showing that cells lacking *CRD1* or the remodeling enzyme *TAZ1* exhibited severely compromised fitness under mitochondrial stress conditions. Together, these findings indicate that increased CL synthesis and remodeling constitute a protective response that stabilizes protein import machineries and enhances mitochondrial protein biogenesis during early stages of mitochondrial dysfunction (Poveda‐Huertes et al., 2021).

Consistent with this model, previous studies have shown that precursor proteins bind more strongly to CL‐containing membranes, which has been proposed to maintain them in a partially unfolded, translocation‐competent state (Endo et al., 1989; Ou et al., 1988; Rietveld et al., 1986; Zardeneta & Horowitz, 1992). Elevated CL levels may therefore further promote efficient protein import under stress conditions. An increase in mitochondrial protein import upon UPR^mt^ induction has also been observed in *C. elegans* (Xin et al., 2022). In organello import assays using artificial Su9‐DHFR and ATFS‐1‐DHFR fusion proteins revealed enhanced import that depended on the activation of the transcriptional response. Transcriptomic analyses further identified increased expression of several subunits of the mitochondrial protein import machinery. However, the study did not determine whether this transcriptional induction indeed results in increased abundance or activity of the corresponding translocase complexes. Moreover, potential changes in mitochondrial membrane lipid composition were not investigated (Xin et al., 2022). Based on the findings in yeast described above, where CL remodeling stabilizes protein import machineries, it is therefore tempting to speculate that lipid changes may likewise contribute to the increased import observed in *C. elegans*. Consistent with a possible conserved role for lipid remodeling, other studies in *C. elegans* reported stress‐induced changes in the mitochondrial lipid composition (Gao et al., 2022), and the MCSR pathway described above was likewise associated with increased CL levels. Together, these observations support a role for CL in the UPR^mt^ in both yeast and nematodes. Whether CL‐dependent stabilization of protein import is conserved in mammalian systems remains an important question for future investigation.

Stress‐induced changes in the mitochondrial lipidome have also been implicated in cellular recovery from mitochondrial dysfunction. Using multiple genetic yeast mutants and chemical treatments to induce the UPR^mt^, a recent study similarly observed increased CL levels and remodeling toward longer and more unsaturated acyl chains following stress induction and further demonstrated that CL is required for efficient recovery from mitochondrial stress (Baker et al., 2025). These findings suggest that stress‐induced increases in CL represent an adaptive response that promotes restoration of mitochondrial function. An interesting observation was a concomitant decrease in triacylglycerol (TAG) levels and lipid droplet abundance. This reduction in storage lipids was not due to impaired lipid droplet biogenesis but rather reflected enhanced mobilization of TAG to support CL biosynthesis, with TAG‐derived acyl groups being redirected toward mitochondrial lipid production. TAG mobilization can occur either through lipolysis, mediated by the TAG lipases Tgl3, Tgl4, and Tgl5, or through lipophagy, in which lipid droplets are delivered to the vacuole for degradation (Fairman & Ouimet, 2022; Kurat et al., 2006). Further analysis demonstrated that TAGs were mobilized predominantly via lipolysis to support CL synthesis, and deletion of *TGL* genes impaired growth recovery at later stages following UPR^mt^ induction. Consistent with a conserved mechanism, experiments in human cell lines confirmed the importance of TAG mobilization for stress recovery and showed that the Tgl homolog ATGL contributes to this process (Baker et al., 2025).

Together, these findings support a model in which mobilization of storage lipids provides acyl groups to fuel mitochondrial lipid biogenesis, particularly CL synthesis, thereby facilitating recovery from mitochondrial stress (Figure 2). Beyond its established role in maintaining respiratory chain function, increased CL levels may also support mitochondrial protein biogenesis during recovery. Stabilization of mitochondrial protein import and assembly machineries is likely to be especially important during the restoration phase, which coincides with renewed cell growth and division and places high demands on mitochondrial protein import capacity.

Taken together, these studies identify mitochondrial lipid remodeling, particularly changes in CL abundance and composition, as an adaptive response to mitochondrial stress. Increased CL levels have been observed upon UPR^mt^ induction in yeast and *C. elegans*, and in both systems, enhanced mitochondrial protein import accompanies the response. In yeast, mechanistic analyses directly linked these observations by demonstrating that CL‐dependent changes in the lipid environment surrounding protein import translocases stabilize their membrane association and promote mitochondrial protein biogenesis. These findings suggest that lipid remodeling represents an early protective response that preserves mitochondrial function during stress. Although the conservation of these mechanisms in mammalian systems remains to be established, the available evidence indicates that stress‐induced modulation of mitochondrial membrane lipids constitutes a previously underappreciated protective component of the UPR^mt^.

## 3 MITOCHONDRIAL MEMBRANE REMODELING WITHIN INTER‐ORGANELLE STRESS NETWORKS

While lipid changes as a protective component of membrane remodeling during the UPR^mt^ are only beginning to emerge, it is well established that the ER increases its size and folding capacity to re‐establish homeostasis during the UPR^ER^ (Bernales et al., 2006; Schuck et al., 2009). This adaptive response is accompanied by a transcriptional program that upregulates lipid biosynthesis, and a clear link between lipid metabolism and the UPR^ER^ has been established over the past decades (Jonikas et al., 2009; Pineau et al., 2009; Promlek et al., 2011; Schuck et al., 2009; Surma et al., 2013; Thibault et al., 2012; Travers et al., 2000). Because the ER is the principal site of lipid synthesis and supplies lipids as building blocks or precursors to other organelles, changes in ER lipid biogenesis pathways are expected to affect membrane composition beyond the ER itself. Consistent with this notion, analysis of the cellular lipidome in yeast following treatment with the commonly used ER stress‐inducing agent dithiothreitol (DTT) revealed mitochondrial‐specific lipid alterations (Reinhard et al., 2020), suggesting that DTT treatment may affect not only the ER but also mitochondria. Treatment with DTT in complete medium for only 1 h resulted in a significant increase in PA, an increase in average acyl chain length and degree of unsaturation, and a modest decrease in CL (Reinhard et al., 2020).

Conversely, mitochondria themselves represent important sites of lipid synthesis and exchange lipids extensively with the ER. ER stress has been shown to affect lipid transport between the ER and mitochondria: Induction of ER stress with DTT resulted in decreased mitochondrial and global phosphatidylcholine (PC) levels and increased mitochondrial phosphatidylethanolamine (PE) levels (Kannan et al., 2016). Although both lipids can be synthesized in the ER, PE can also be generated in mitochondria and endosomes and subsequently transported to the ER, where it serves as a precursor for PC synthesis. ER stress reduced this PE‐to‐ER transport, providing a mechanistic explanation for the observed decrease in PC and increase in PE levels (Kannan et al., 2016). These findings demonstrate that ER stress can reshape mitochondrial lipid composition by altering inter‐organelle lipid trafficking.

**FIGURE 3:**
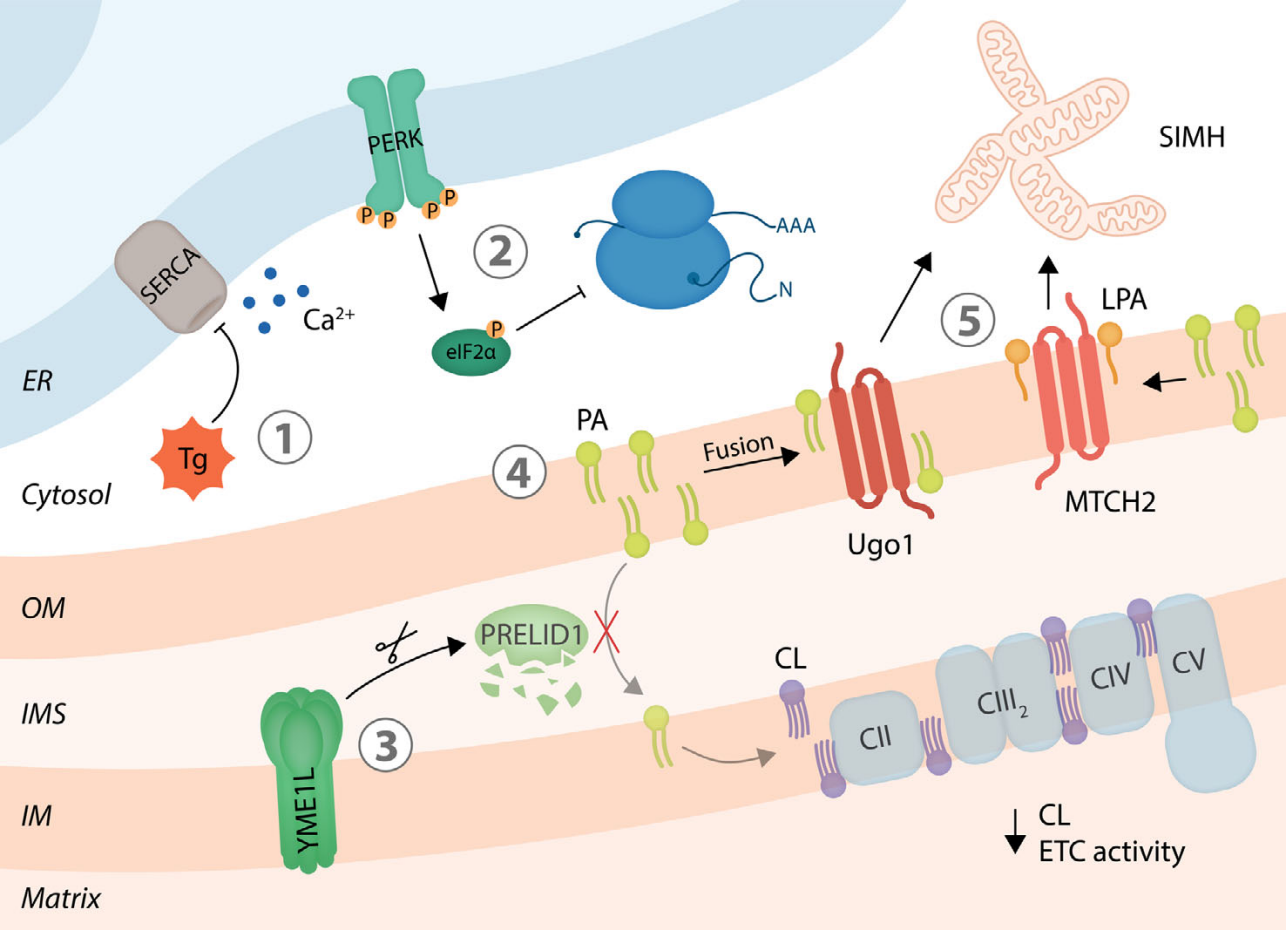
Schematic of the consequences of endoplasmic reticulum (ER) stress on mitochondria. Thapsigargin (Tg) inhibits the ER‐resident Ca^2+^ ATPase SERCA (1), thereby inducing the unfolded protein response of the endoplasmic reticulum (UPR^ER^). Consequently, the ER stress sensor PERK is activated and phosphorylates eIF2α, resulting in attenuated translation (2). Constitutive YME1L‐mediated degradation of the phosphatidic acid (PA) transporter PRELID1 (3) causes an accumulation of PA in the mitochondrial outer membrane (OM) and decreased cardiolipin (CL) synthesis, thus reducing electron transport chain (ETC) activity (4). PA accumulation is followed by recruitment of the OM fusion machinery protein Ugo1 (5). Similarly, its human functional homolog MTCH2 senses elevated levels of lysophosphatidic acid (LPA) and promotes protective mitochondrial fusion (stress‐induced mitochondrial hyperfusion [SIMH]).

While these studies demonstrate that ER stress alters mitochondrial lipid composition through changes in lipid transport in yeast, more recent work in mammalian systems has revealed a direct link between UPR^ER^ signaling and mitochondrial membrane remodeling through regulation of specific mitochondrial phospholipids (Figure 3). Although the impact of ER stress on mitochondrial quality control pathways is well established (Rainbolt et al., 2014; Verfaillie et al., 2012), its role in regulating mitochondrial membrane composition represents a more recent development and is mediated through the PERK signaling axis (Lebeau et al., 2018; Perea et al., 2023). ER stress activates the ER‐resident kinase PERK, which phosphorylates eIF2α, resulting in translational attenuation and activation of the transcription factor ATF4. Among multiple downstream targets, ATF4 promotes transcription of mitochondrial chaperones and proteases (Quiros et al., 2015; Rainbolt et al., 2014). Using mouse embryonic fibroblasts (MEFs) treated with thapsigargin, an inhibitor of the ER calcium transporter SERCA, time‐resolved analyses revealed sequential changes in mitochondrial morphology (Lebeau et al., 2018). Following an initial phase of mitochondrial fragmentation, the mitochondrial network underwent prolonged elongation, which was later followed by renewed fragmentation upon extended treatment, likely reflecting apoptotic signaling. These observations demonstrate that ER stress influences mitochondrial morphology in a temporal manner dependent on stress duration, reminiscent of stage‐specific membrane changes observed during UPR^mt^ induction (Poveda‐Huertes et al., 2021). The phase of mitochondrial elongation corresponds to stress‐induced mitochondrial hyperfusion (SIMH), a response previously described under various stress conditions, including starvation, ribosome inhibition, and ER stress (Gomes et al., 2011a; 2011b; Sabouny et al., 2017; Tondera et al., 2009). Thapsigargin‐induced SIMH was prevented by pharmacological inhibition of PERK‐dependent eIF2α phosphorylation and was absent in PERK‐deficient MEFs but did not depend on ATF4‐mediated transcription, indicating a non‐transcriptional PERK‐dependent mechanism (Lebeau et al., 2018).

Previous studies had shown that PERK also influences mitochondrial protein quality control by promoting degradation of the TIM23 subunit TIM17A via the IM AAA protease YME1L (Rainbolt et al., 2013). Testing a potential role for YME1L in SIMH revealed that YME1L depletion reduced mitochondrial elongation following thapsigargin treatment. To elucidate the molecular basis of PERK‐induced SIMH, mitochondrial PA levels were examined (Perea et al., 2023). Accumulation of PA in the OM has been shown to promote mitochondrial elongation, and PERK activation was previously reported to increase cellular PA levels during ER stress (Bobrovnikova‐Marjon et al., 2012). Consistent with this, mass spectrometric analysis revealed increased mitochondrial PA levels following ER stress.

Transport of PA from the OM to the IM in mammalian cells is mediated by PRELID1, a substrate of YME1L, linking these observations. PRELID1, like TIM17A, exhibits a short half‐life, and PERK‐dependent translational attenuation may therefore promote its rapid depletion by degradation via YME1L. Indeed, PRELID1 levels rapidly decreased following thapsigargin treatment in MEFs but remained stable in YME1L‐deficient cells. Reduced PA transport to the IM is expected to limit CL synthesis, as PA serves as its precursor, and accordingly, decreased CL levels were observed in thapsigargin‐treated cells. However, depletion of PRELID1 alone did not induce mitochondrial hyperfusion, indicating that impaired PA transport across the IMS is not sufficient to explain PA‐mediated morphological changes, but that additional mechanisms contribute to PA accumulation. PERK has been reported to possess intrinsic lipid kinase activity capable of generating PA from diacylglycerol (Bobrovnikova‐Marjon et al., 2012), providing a second source for increased PA levels. How PA accumulation in the OM promotes SIMH remains to be fully elucidated. Inhibition of DRP1‐dependent mitochondrial fission has been proposed, but PA has also been identified as the first lipid directly involved in the biogenesis of a mitochondrial OM protein, Ugo1, a component of the mitochondrial fusion machinery in yeast (Vögtle et al., 2015). PA was sufficient to promote Ugo1 import and assembly in protein‐free liposomes mimicking the OM lipid composition, and elevated PA levels enhanced Ugo1 import in vivo. Similarly, the human OM protein MTCH2, related to Ugo1, mediates starvation‐induced mitochondrial hyperfusion through a mechanism dependent on lysophosphatidic acid (LPA) and was found to interact with enzymes that convert LPA to PA (Labbe et al., 2021). Further studies will be required to determine how PA accumulation drives mitochondrial elongation and whether these mechanisms are conserved across species.

While ER stress has well‐established effects on mitochondrial function, and while these increasing mechanistic insights support its role in mitochondrial membrane remodeling, the consequences of dysfunction in other organelles for mitochondrial physiology remain less well understood. A recent study addressed this question by performing a genome‐wide CRISPR screen to identify mitochondrial stress inducers in human CAL51 breast cancer cells (Lee et al., 2024). Increased expression of the mitoribosomal protein MRPL12 was used as a readout of mitochondrial stress and was detected for approximately 30% of RNA guides. After excluding genes encoding mitochondrial proteins and narrowing candidates by gene ontology analysis, the study focused on three proteins: the peroxisomal import protein PEX26, the Golgi organization factor GOLGA5, and the ER protein import regulator and stress responder SEC62. Disruption of each gene resulted in pronounced fragmentation of the mitochondrial network and altered mitochondrial association with the respective organelle (Lee et al., 2024).

High‐resolution focused ion beam scanning electron microscopy (FIB‐SEM) revealed that all three knockouts exhibited similar changes in mitochondrial size and morphology. These structural analyses were complemented by comprehensive‐omics approaches, which showed minimal overlap in transcriptomic changes but greater similarity at the proteomic level, with approximately 40% of detected proteins significantly altered across all knockout cell lines. Global lipidomic analyses identified widespread lipid alterations, although detailed characterization of mitochondrial lipid species remains to be performed and would provide valuable insight into the observed morphological changes (Lee et al., 2024). The present descriptive findings also raise the question of whether mitochondrial defects arise from disrupted inter‐organelle biogenesis networks or from direct effects on mitochondrial function. Consistent with the latter possibility, previous work showed that loss of peroxins required for peroxisomal protein import leads to mislocalization of peroxisomal proteins to the mitochondrial OM in yeast (Nuebel et al., 2021; Vögtle, 2021). Similar protein mistargeting was observed in fibroblasts from Zellweger syndrome patients lacking PEX3, resulting in localization of peroxisomal proteins to mitochondria and likely contributing to altered mitochondrial morphology (Nuebel et al., 2021). These observations suggest that mitochondrial changes can arise from both altered inter‐organelle communication and direct protein mistargeting. While the current high‐resolution imaging of the knockout cell lines has provided valuable initial insights, detailed mechanistic analysis of these novel stress models will be required to shed light on the molecular basis of inter‐organelle effects on mitochondrial architecture and function.

Taken together, increasing evidence indicates that mitochondria respond to stress originating in other cellular organelles not only through changes in mitochondrial protein quality control but also through membrane remodeling. ER stress, in particular, reshapes mitochondrial lipid composition, influences phospholipid metabolism, and alters mitochondrial morphology through defined signaling pathways. More broadly, emerging studies suggest that mitochondrial membrane dynamics are embedded within inter‐organelle stress response networks, highlighting membrane remodeling as a central component of cellular adaptation to organelle dysfunction.

## 4 DISCUSSION

Mitochondrial membrane remodeling, particularly through regulated changes in CL, is emerging as a protective branch of the UPR^mt^. Rather than representing a passive consequence of organelle dysfunction, lipid remodeling appears to constitute an adaptive mechanism that supports mitochondrial protein biogenesis, preserves bioenergetic function, and facilitates recovery from stress. However, many fundamental questions remain unresolved. Future studies should systematically analyze lipid alterations induced by distinct UPR^mt^ triggers to determine whether common lipid signatures exist or whether specific stressors generate unique remodeling patterns. Comparative analyses across early and late stages of mitochondrial dysfunction will be particularly informative to define when lipid remodeling acts as a reversible adaptive response and when it transitions toward irreversible outcomes such as mitophagy or cell death. Understanding the temporal dynamics, amplitude, and reversibility of stress‐induced lipid changes will be essential to define their functional relevance.

Beyond mitochondria‐intrinsic responses, it will be important to clarify how lipid remodeling is coordinated within inter‐organelle stress networks. The extent to which ER stress, peroxisomal dysfunction, or other organelle perturbations shape mitochondrial membrane composition—and whether these effects are protective or not—remains largely unexplored. Dissecting these communication pathways will provide insight into how cells integrate organelle stress signals at the level of membrane architecture. Determining the evolutionary conservation of lipid‐mediated stress adaptation across species will further clarify whether membrane remodeling represents a fundamental principle of organelle quality control.

Addressing these questions will also depend on methodological challenges. In particular, quantitative and spatially resolved lipidomics will be instrumental in resolving organelle‐specific lipid dynamics during stress and in identifying local changes in membrane composition that cannot be captured by bulk analyses. Different lipid profiles of the mitochondrial outer and inner membranes and distinguishing the two different leaflets present an additional technical challenge. However, these analyses may enable the identification of shared and stress‐specific mitochondrial “lipid fingerprints”, which could serve as biomarkers of mitochondrial stress states and reveal previously unrecognized regulatory mechanisms.

Finally, a deeper mechanistic understanding of lipid‐mediated stress adaptation may open new therapeutic opportunities. Targeting lipid metabolic pathways that support mitochondrial adaptation, such as CL remodeling or storage lipid mobilization, may provide novel strategies to enhance mitochondrial resilience in disease contexts. Given the central role of mitochondrial dysfunction in aging and numerous human pathologies, defining lipid‐dependent protective mechanisms represents an important avenue for future research.

## AUTHOR CONTRIBUTIONS

**Carlotta Peselj:** Conceptualization; writing – original draft. **F.‐Nora Vögtle:** Conceptualization; funding acquisition; writing – review and editing; supervision. **Lena J. Reichert:** Conceptualization; visualization.

## Data Availability

Data sharing not applicable to this article as no datasets were generated or analyzed during the current study.
